# A Phase I study of the pharmacokinetics (PK), bioavailability, dose proportionality, safety, and tolerability of topiramate injection compared to Oral topiramate in healthy adult participants

**DOI:** 10.1002/epi.70432

**Published:** 2026-08-10

**Authors:** Adeboye O. Bamgboye, James C. Cloyd, Bernard D. King, Lisa D. Coles

**Affiliations:** ^1^ Department of Experimental and Clinical Pharmacology, College of Pharmacy University of Minnesota Minneapolis Minnesota USA; ^2^ Center for Orphan Drug Research, College of Pharmacy University of Minnesota Minneapolis Minnesota USA; ^3^ Macnas Consulting International, Inc. New Bern North Carolina USA

**Keywords:** Anti‐seizure medication, bridging therapy, clinical trial, epilepsy, topiramate

## Abstract

This Phase I study evaluated the safety, tolerability, and pharmacokinetics of a 30‐min topiramate injection (intravenous [IV] TPM) infusion compared with oral topiramate (oral TPM) in healthy adult participants. In this randomized, open‐label, dose escalation, crossover study with 37 healthy participants received single doses of 50, 100, or 200 mg of an oral TPM tablet and an IV TPM formulation, using sulfobutylether beta‐cyclodextrin as a solubilizer and stabilizer, infused over 30 min, with a 12‐day washout. Blood samples for pharmacokinetic assessments were collected at predose through 192 h post dose. Concentration–time data were analyzed using non‐compartmental analysis, and IV TPM relative bioavailability was determined. Topiramate injection AUC_inf_ (total exposure) increased linearly with dose. The mean (90% confidence interval [CI]) relative bioavailability was 97.1% (94.6–99.7%). The mean (± SD) clearance for 50, 100, and 200 mg IV TPM was 1.4 L/h (±0.2), 1.3 L/h (±0.2), and 1.3 L/h (±0.2), respectively. Half‐life was comparable across routes. Dose proportionality was demonstrated for AUC_inf_ over the 50–200 mg range. No serious adverse events were reported. These findings suggest that when oral TPM administration is not feasible, equivalent doses of topiramate injection infused over 30 min at intervals can temporarily replace oral dosing until it can be resumed.

**Key points**

- Our results show that topiramate (TPM) injection administered as a 30‐min infusion is safe and well tolerated in healthy adult participants.
- There were no serious adverse events. Treatment‐emergent adverse events were mild and included headache, dizziness, and paresthesia.
- Topiramate injection has a relative bioavailability of ~100% compared to oral topiramate, and clearance is constant across the 50–200 mg dose range.
- For patients unable to take oral TPM, equivalent doses of TPM injection can be infused over 30 min as a temporary replacement.

## 1 INTRODUCTION

Epilepsy is a complex neurological condition affecting over 70 million people worldwide.^1^, ^2^ It is characterized by recurrent seizures resulting from a complicated interplay of neurobiological, cognitive, psychosocial, and social factors.^3^, ^4^, ^5^, ^6^

The mainstay of epilepsy treatment is anti‐seizure medications (ASMs), which work via diverse mechanisms to prevent or terminate seizures.^7^ Topiramate (TPM) is an ASM approved for use as monotherapy or adjunctive therapy for partial‐onset seizures, primary seizures, and seizures associated with Lennox–Gastaut syndrome in adults and pediatric patients (2 to 16 years). TPM is currently available as immediate‐release tablets, extended‐release capsules, sprinkle capsules, and as an oral solution.

Stable ASM concentrations are essential for optimal seizure control. When oral administration is not feasible, such as during surgery or loss of consciousness, intravenous (IV) administration is required to maintain drug concentrations above seizure thresholds. There are no approved intravenous TPM options for patients.^8^ This lack of parenteral TPM formulations could lead to undesirable consequences from missed doses, such as breakthrough seizures. TPM injection is an intravenous formulation of TPM dissolved in sulfobutylether beta‐cyclodextrin, (CyDex Pharmaceuticals, Inc.) being developed as a replacement therapy where oral administration is not feasible. The cyclodextrin matrix has been used in many approved parenteral products since 2002, including ASMs and, more recently, VEKLURY (remdesivir), an injection approved in the United States for the treatment of coronavirus disease 2019 (COVID‐19) in adults and pediatric patients (birth to <18 years and weighing ≥1.5 kg).^9^, ^10^

The primary study objective was to determine the pharmacokinetics (PK), including bioavailability, of TPM injection relative to oral TPM following a single dose in healthy participants to inform clinical use. The secondary objectives were to: (1) assess dose proportionality, and (2) compare the safety and tolerability of TPM injection administered as a 30‐min intravenous infusion with oral TPM in healthy participants.

## 2 MATERIALS AND METHODS

### 2.1 Participants

Eligible participants were required to be 18 years or older, with a body mass index (BMI) ≥18.5 and ≤32.0 kg/m^2^ and weight between 110 and 230 pounds (49.9–104.3 kg) at screening. Key exclusion criteria included a history of seizures or migraine, renal or hepatic impairment, and a history of glaucoma or increased intraocular pressure.

### 2.2 Study drug

The study drug was formulated as a 10 mg/mL solution with 100 mg/mL sulfobutylether beta‐cyclodextrin (Captisol, CyDex Pharmaceuticals, Inc.) in a 50 mM phosphate buffer. The formulation was supplied as 10 mL of TPM injection in a 10 cc glass vial. The comparator was Topamax 50 mg, 100 mg, and 200 mg tablets (Janssen Pharmaceuticals, Inc.). Full formulation details have been described previously.^8^

### 2.3 Study design

This was a Phase 1, randomized, single‐center, open‐label, crossover, dose escalation study comparing TPM injection administered intravenously over 30 min to Topamax tablets administered as a single dose. The study was conducted at Medpace (Cincinnati, Ohio) under IND #153216, approved by the Advarra Institutional Review Board, and conducted in accordance with the Declaration of Helsinki and Good Clinical Practice Guidelines.

The study was conducted in three phases in 37 healthy participants across three sequential dose cohorts (50, 100, and 200 mg; *n* = 11–13 per cohort). Each phase included screening, dosing Period 1, washout, dosing Period 2, and follow‐up. Each cohort was randomized 1:1 to receive TPM injection (30‐min intravenous [IV] infusion) or Topamax tablet in dosing period 1(Day 0), crossing over to the alternative formulation in dosing Period 2 (Day 21), following a washout (Day 8–Day 20). Both doses were administered after an overnight fast. After completion of the 50 mg phase, a second group received 100 mg of the formulations. Dose escalation to 200 mg proceeded following a Safety Review Committee (SRC) evaluation of safety, tolerability, and available PK data from the preceding cohorts. PK sampling and safety assessments were conducted through Day 28, with a final safety follow‐up on Day 30. Full study design details, are provided in the Supplementary Materials.

### 2.4 Pharmacokinetic evaluation and analysis

Blood samples for pharmacokinetic assessments were collected pre‐dose and 0.5, 1, 1.5, 2, 3, 4, 6, 8, 12, 16, 24, 36, and 48 h (±10 min) after the start of drug administration, as well as 72, 96, 120, 144, and 192 h (±60 min) during outpatient visits.

A full description of the pharmacokinetic analysis is contained in the Supplementary Materials.

### 2.5 Statistical methods

Based on data available regarding administration of TPM, 36 participants (12 per dose group) were expected to be sufficient to determine the pharmacokinetics, bioavailability, and dose proportionality of TPM injection compared to oral TPM.

The geometric means, arithmetic means, geometric mean ratios, and 90% confidence intervals (CIs) were calculated for the maximum plasma concentration (C_max_), area under the concentration–time curve from zero to infinity (AUC_0‐inf_), and from zero to time t (AUC_0‐t_).

To assess dose proportionality (DP), a power model was fitted to log‐transformed pharmacokinetic parameters (C_max_, AUC_0‐inf_, and AUC_0‐t_).^11^ Full statistical methods are provided in the Supplementary Materials.

### 2.6 Safety evaluation

TPM injection safety was evaluated by assessing the incidence of treatment‐emergent adverse events (TEAEs), serious adverse events, and withdrawals from randomization through the end of study, as well as changes in clinical laboratory values. A full description of the safety evaluation is contained in the Supplementary Materials.

## 3 RESULTS

### 3.1 Study subjects

Thirty‐seven healthy volunteers (10 female, 27 male) were included in the analysis. The mean (SD) weight for the participants was 79.9 (13.3) kg, and the mean (SD) age was 46.1 (11) years. The baseline characteristics of the studied population are provided in Table S1.

### 3.2 Pharmacokinetics and bioavailability results

A total of 1291 unique plasma topiramate concentrations were used for PK analysis. The summary of the pharmacokinetic parameters is presented in Table 1. The C_max_ and AUC values increased with dose for both routes of administration. Mean maximum plasma concentration (C_max_) ranged from 857 to 5417 ng/mL (0.9–5.4 μg/mL) for TPM injection and 725 ng/mL to 4183 ng/mL (0.7–4.2 μg/mL) for Topamax across the dose range. Clearance (CL), volume of distribution (Vd), and elimination half‐life values were comparable across doses and routes of administration (Table 1). The geometric mean dose‐normalized AUC_inf_ (total exposure) ratio was 103% (90% CI: 100–106), indicating that oral bioavailability was ~100%. Exploratory analysis of the relationship between PK parameters and dose identified significant dose‐related changes in the apparent volume of distribution (Vd/F) and half‐life (Table S3).

**TABLE 1:** Summary of pharmacokinetic parameters following a 30‐min single‐dose infusion of intravenous topiramate and oral topiramate in healthy participants.

PK parameter	Route	Dose groups		
		50 mg (*n* = 13^a^/11^b^)	100 mg (*n* = 12^a^/11^b^)	200 mg (*n* = 12^a^/12^b^)
C_max_ (ng/mL)	IV	857 ± 190	2514 ± 785	5417 ± 818
	Oral	725 ± 243	2040 ± 568	4183 ± 962
AUC_last_ (h.ng/mL)	IV	31 651 ± 5737	74 219 ± 13 005	154 662 ± 28 378
	Oral	32 353 ± 6255	79 158 ± 14 106	161 683 ± 31 043
T_max_ (h)	IV	0.5 (0.5–1.0)	0.5 (0.5–0.5)	0.5 (0.5–0.5)
	Oral	1.4 (0.5–4.0)	1.2 (0.5–2.0)	1.3 (0.5–3.0)
AUC_inf_ (h.ng/mL)	IV	33 991 ± 11 958	80 496 ± 13 447	162 797 ± 29 133
	Oral	37 582 ± 7007	86 121 ± 14 628	169 561 ± 32 492
Half‐Life (h)	IV	72 ± 24.7	63 ± 7.0	53 ± 6.6
	Oral	78 ± 11.9	65 ± 9.7	51 ± 4.6
CL (CL/F) (L/h)	IV	1.4 ± 0.2	1.3 ± 0.2	1.3 ± 0.2
	Oral	1.4 ± 0.2	1.2 ± 0.2	1.2 ± 0.2
Vd (Vd/F) (L/kg)	IV	2.3 ± 0.5	1.7 ± 0.4	1.4 ± 0.4
	Oral	2.2 ± 0.5	1.6 ± 0.5	1.3 ± 0.3
F_rel_ (%)*		97.1 (94.6–99.7)		
F_abs_ (%)*		103 (100.3–105.8)		

*Note*: Data are presented as arithmetic mean ± standard deviation except for T_max_ (median [range]) and F_**rel**_ / F_**abs**_ (%).

Abbreviations: AUC_inf_, area under the plasma concentration–time curve from time 0 to infinity; AUC_last_, area under the plasma concentration–time curve from time 0 to the last sampling time; CI, confidence interval; CL/F, apparent clearance; CL, clearance; C_max_, maximum observed drug concentration; F_abs_, absolute bioavailability; F_rel_, relative bioavailability; IV, intravenous; T_max_, time to maximum plasma concentration; Vd/F, apparent volume of distribution; Vd, volume of distribution.

* Expressed as the geometric mean of the dose‐normalized AUC_inf_ ratios with 90% CIs.

^a^ IV administration sample sizes.

^b^ Oral administration sample sizes.

### 3.3 Dose proportionality

The slope parameter (β1) estimates and 90% CIs for C_max_ and AUC_last_ exceeded the pre‐specified acceptance range (0.839–1.161), indicating supra‐proportional increases with dose for these parameters. In contrast, the 90% CI for β1 for the AUC_inf_ fell within the acceptance range (0.839–1.161), demonstrating dose proportionality over the 50–200 mg dose range. Detailed results are presented in Table S2.

### 3.4 Safety

TPM injection was generally well tolerated. TEAE counts (any TEAEs) were similar between the oral and intravenous dose groups at the 50 mg and 200 mg dose levels, whereas participants experienced more TEAEs following the 100 mg infusion than the 100 mg oral tablet (7 vs 4). Study‐related TEAEs were more prevalent in the 200 mg dose (Table 2). No severe or life‐threatening TEAEs were observed for either the oral or intravenous formulations. Four individuals experienced TEAEs with moderate maximum severity: 2 in the 50 mg IV group, 1 in the 100 mg IV group, and 1 in the 200 mg oral group. An overall summary of the frequently reported TEAEs by system organ class is provided in Table S4. Nervous system–related TEAEs, including paresthesia, dizziness, and headache, were the most frequently occurring. Three individuals discontinued the study due to TEAEs.

**TABLE 2:** Overview of AEs after oral and intravenous topiramate.

	Cohort 1 *(50 mg IV)* (*N* = 13)	Cohort 2 *(50 mg oral)* (*N* = 11)	Cohort 3 *(100 mg IV)* (*N* = 12)	Cohort 4 *(100 mg oral)* (*N* = 11)	Cohort 5 (200 mg IV) (*N* = 12)	Cohort 6 (200 mg oral) (*N* = 12)	Total (*N* = 37)
	*n* (%) e	*n* (%) e	*n* (%) e	*n* (%) e	*n* (%) e	*n* (%) e	*n* (%) e
Any TEAEs	2 (15.4) 5	2 (18.2) 2	7 (58.3) 15	4 (36.4) 7	7 (58.3) 11	8 (66.7) 14	21 (56.8) 54
Any TEAEs by maximum severity							
Mild	0 (0.0)	2 (18.2)	6 (50.0)	4 (36.4)	7 (58.3)	7 (58.3)	17 (45.9)
Moderate	2 (15.4)	0 (0.0)	1 (8.3)	0 (0.0)	0 (0.0)	1 (8.3)	4 (10.8)
Severe	0 (0.0)	0 (0.0)	0 (0.0)	0 (0.0)	0 (0.0)	0 (0.0)	0 (0.0)
Life‐threatening	0 (0.0)	0 (0.0)	0 (0.0)	0 (0.0)	0 (0.0)	0 (0.0)	0 (0.0)
Any TEAEs by study drug relation							
Related	0 (0.0)	0 (0.0)	4 (33.3)	1 (9.1)	7 (58.3)	8 (66.7)	13 (35.1)
Possibly related	1 (7.7)	0 (0.0)	2 (16.7)	0 (0.0)	0 (0.0)	0 (0.0)	3 (8.1)
Unlikely related	1 (7.7)	0 (0.0)	0 (0.0)	1 (9.1)	0 (0.0)	0 (0.0)	2 (5.4)
Not related	0 (0.0)	2 (18.2)	1 (8.3)	2 (18.2)	0 (0.0)	0 (0.0)	3 (8.1)
Any study drug‐related TEAEs	0 (0.0) 0	0 (0.0) 0	4 (33.3) 8	1 (9.1) 1	7 (58.3) 10	8 (66.7) 13	13 (35.1) 32
Any study drug‐related TEAEs by maximum severity							
Mild	0 (0.0)	0 (0.0)	3 (25.0)	1 (9.1)	7 (58.3)	7 (58.3)	11 (29.7)
Moderate	0 (0.0)	0 (0.0)	1 (8.3)	0 (0.0)	0 (0.0)	1 (8.3)	2 (5.4)
Severe	0 (0.0)	0 (0.0)	0 (0.0)	0 (0.0)	0 (0.0)	0 (0.0)	0 (0.0)
Life‐threatening	0 (0.0)	0 (0.0)	0 (0.0)	0 (0.0)	0 (0.0)	0 (0.0)	0 (0.0)
Any TESAEs	0 (0.0) 0	0 (0.0) 0	0 (0.0) 0	0 (0.0) 0	0 (0.0) 0	0 (0.0) 0	0 (0.0) 0
Any TEAEs leading to discontinuation of study	2 (15.4) 2	0 (0.0) 0	1 (8.3) 1	0 (0.0) 0	0 (0.0) 0	0 (0.0) 0	3 (8.1) 3
Any TEAEs leading to death	0 (0.0) 0	0 (0.0) 0	0 (0.0)	0 (0.0) 0	0 (0.0) 0	0 (0.0) 0	0 (0.0) 0
Any frequently reported TEAEs	1 (7.7) 2	1 (9.1) 1	6 (50.0) 10	4 (36.4) 5	7 (58.3) 9	6 (50.0) 8	17 (45.9) 35

*Note*: % = 100 × *n/N* where *n* = the number of participants with one or more TEAEs. e = number of TEAEs in the cohort. Note that the number of events is not presented for summaries by maximum severity or drug relation. TEAEs were defined as AEs that started after the first dose of the study drug. Frequently reported TEAEs were defined as TEAEs that had an incidence of at least 5% of the total subject population. AE‐reported terms were coded using the MedDRA version 26.1.

Abbreviations: AE, adverse event; MedDRA, Medical Dictionary for Regulatory Activities; TEAE, treatment‐emergent adverse event; TESAE, treatment‐emergent serious adverse event.

## 4 DISCUSSION

In that there are no parenteral TPM formulations, patients unable to take TPM by mouth face the consequences of abrupt therapy discontinuation, which underscores the need for an injectable product. In this comprehensive Phase I study, TPM injection total exposures increased proportionally with dose, consistent with a previous study of intravenous TPM pharmacokinetics in healthy adults.^8^

The relative bioavailability of TPM injection was 97.1% (90% CI: 94.6–99.7), and the absolute bioavailability estimate of oral TPM was 103% (90% CI: 100.3–105.8), which is consistent with reported results from Clark et al.^8^ Taken together, these results indicate comparable systemic exposure following the same oral and intravenous doses, which has clinical implications when oral therapy is interrupted, and bridging therapy is warranted. As an example, given an oral bioavailability of ~100%, a patient on 100 mg of oral TPM who is switched to 100 mg of IV TPM, will have the same systemic exposure when taking the oral dose. Our results show that a 30‐min infusion of IV TPM achieved C_max_ values comparable to equivalent doses of oral TPM. Given that infusion duration affects the peak concentrations attained, shorter infusions would increase C_max_ values, whereas prolonged infusions would lower peak concentrations. The relatively rapid infusion rate without serious adverse effects supports the use of TPM injection in replacement therapy for emergent conditions such as status epilepticus and short‐term oral‐to‐IV bridging.

Clearance estimates were constant across doses, suggesting dose‐independent CL. In contrast, half‐life, although similar between routes at the same doses, was shorter at higher doses. We hypothesize that the reported saturable binding of TPM to carbonic anhydrase in red blood cells (RBCs), which could lead to lower Vd at higher doses, accounts for this observation.^12^ Similarly, assay sensitivity can affect the half‐lives,^13^ potentially contributing to observable differences.

Clark et al.^8^ reported shorter half‐lives following 50 and 100 mg oral and IV doses; other studies report half‐life estimates overlapping with ours.^14^, ^15^ Although we observed dose‐related half‐life differences, oral and IV half‐lives were similar at the same dose, suggesting that dosing‐interval adjustments are not needed when switching to IV. Given the intended short‐term use (hours to days) and the absence of any serious adverse events, safety concerns with oral‐to‐IV bridging are not expected.

Dose proportionality analysis showed that AUC_inf_ was dose‐proportional using pre‐specified criteria, but C_max_ exceeded the upper limit, likely due to considerable inter‐individual variability in C_max_.

Although C_max_ following a 30‐min infusion was ~18%–30% higher than that after the same oral dose, the adverse events frequency were similar at the highest dose. Furthermore, the concentrations attained were consistent with recommended concentrations.^16^, ^17^

TPM injection was generally well tolerated. The TEAEs observed were predominantly nervous system‐related and consistent with previous reports.^8^, ^14^, ^18^ This study extends previous safety findings^8^ by including a higher 200 mg dose. Taken together, the overall safety profile of TPM injection appears acceptable.

Study limitations include the small sample size, which limits characterization of the frequency of adverse events associated with IV TPM and an inability to evaluate the potential effect of age and renal function on the pharmacokinetics and safety of TPM injection.

In conclusion, TPM injection demonstrated a favorable safety profile, dose‐proportional total systemic exposure, and has a relative bioavailability of ~100%.

## AUTHOR CONTRIBUTIONS

**Adeboye O. Bamgboye:** Conceptualization, Writing–original draft, Visualization, Methodology, Writing – review & editing. **James C. Cloyd**: Conceptualization, Writing – original draft, Writing – review & editing, Supervision. **Bernard D. King**: Data Curation, Conceptualization, Writing – review & editing, Study Oversight. **Lisa D. Coles**: Conceptualization, Writing – original draft, Writing–review & editing, Methodology, Supervision. All authors approved the final version.

## FUNDING INFORMATION

This clinical trial was funded by CURx Pharmaceuticals. The subsequent data analysis and manuscript preparation were not supported by any specific funding.

## CONFLICT OF INTEREST STATEMENT

Dr. Cloyd is entitled to royalties under a licensing agreement between Ligand Pharmaceuticals, Inc., and the University of Minnesota pertaining to injectable topiramate. This relationship has been reviewed and managed by the University of Minnesota in accordance with its Conflict‐of‐Interest policies. Dr. King is Chief Medical Officer at CURx Pharmaceuticals, which provided funding for the Phase 1 trial described in this manuscript.

## ETHICS STATEMENT

The study protocol was reviewed and approved by the Advarra Institutional Review Board prior to study initiation. The study was conducted under IND #153216 in accordance with the Declaration of Helsinki, applicable local regulations, and in compliance with Good Clinical Practice Guidelines.

## ETHICAL PUBLICATION STATEMENT

We confirm that we have read the Journal's position on issues involved in ethical publication and affirm that this report is consistent with those guidelines.

## Supporting information


**Figure S1.** Study Design Schematic. ADM, admission to study unit; D/C, discharge from study unit; EOS, end of study; FU, follow‐up; RDM, randomization; TPM, topiramate formulation (injection or tablet); TPM2, alternate topiramate formulation; V, out‐patient visit to study unit.
**Figure S2.** Mean plasma concentration–time profiles of topiramate in healthy volunteers following intravenous (A) and oral (B) administration. Error bars represent the standard error. Blue, red, and green lines represent 200 mg, 100 mg, and 50 mg doses, respectively.
**Table S1.** Participant characteristics.
**Table S2.** Dose proportionality analysis results.
**Table S3.** Results of two‐way analysis of variance (ANOVA) evaluating the effects of dose (50, 100, 200 mg), route (intravenous [IV] vs oral), and dose–route interaction on selected pharmacokinetic parameters.
**Table S4.** Frequently reported TEAEs by SOC and PT – safety population.

## Data Availability

Individual participant data supporting the results presented in this study are not publicly available as they have been submitted to the U.S. Food and Drug Administration and are subject to licensing agreements. Aggregate data may be made available upon reasonable request with approval from the sponsor.
