# Supplementary material for: A Phase I study of the pharmacokinetics (PK), bioavailability, dose proportionality, safety, and tolerability of topiramate injection compared to Oral topiramate in healthy adult participants
Source: Epilepsia. 2026 Aug 10;67(10):e178–84. doi: 10.1002/epi.70432 (PMC13675767; doi:10.1002/epi.70432)
Supplement: Supplementary file 1 [file EPI-67-e178-s001.docx]

**Title:** A Phase I Study of the Pharmacokinetics (PK), Bioavailability, Dose Proportionality, Safety, and Tolerability of Topiramate Injection Compared to Oral Topiramate in Healthy Adult Participants

**Authors:** Adeboye O. Bamgboye^1,2^, James C. Cloyd^1,2^, Bernard D.King^3^, Lisa D. Coles^1,2^

**SUPPORTING INFORMATION**

**Figure S1**. Study Design Schematic. ADM: Admission to study unit; RDM: Randomization; TPM: Topiramate formulation (Injection or Tablet); D/C: Discharge from study unit; V: out-patient visit to study unit; TPM2: Alternate topiramate formulation; FU: Follow-up; EOS: End of Study.

**Figure S2.** Mean plasma concentration-time profiles of topiramate in healthy volunteers following intravenous (A) and oral (B) administration. Error bars represent the standard error. Blue, red, and green lines represent 200 mg, 100 mg, and 50 mg doses, respectively.

**Table S1.** Participant Characteristics

| Characteristics  Statistic/  Category | Cohort 1  (N=7) | Cohort 2  (N=6) | Cohort 3  (N=6) | Cohort 4  (N=6) | Cohort 5  (N=6) | Cohort 6  (N=6) | Total  (N=37) |
| --- | --- | --- | --- | --- | --- | --- | --- |
| Age (years) | | | | | | | |
| n | 7 | 6 | 6 | 6 | 6 | 6 | 37 |
| Mean | 43.9 | 43.5 | 41.8 | 54.8 | 48.3 | 44.5 | 46.1 |
| Standard deviation | 10.19 | 10.41 | 14.43 | 7.03 | 8.78 | 12.76 | 10.98 |
| Sex, n (%) | | | | | | | |
| Male | 5 (71.4) | 6 (100.0) | 5 (83.3) | 3 (50.0) | 6 (100.0) | 2 (33.3) | 27 (73.0) |
| Female | 2 (28.6) | 0 (0.0) | 1 (16.7) | 3 (50.0) | 0 (0.0) | 4 (66.7) | 10 (27.0) |
| Childbearing potential, n (%)[1] | | | | | | | |
| Yes | 1 (50.0) | 0 (0.0) | 1 (100.0) | 0 (0.0) | 0 (0.0) | 2 (50.0) | 4 (40.0) |
| No | 1 (50.0) | 0 (0.0) | 0 (0.0) | 3 (100.0) | 0 (0.0) | 2 (50.0) | 6 (60.0) |
| Race, n (%) | | | | | | | |
| White | 5 (71.4) | 2 (33.3) | 4 (66.7) | 6 (100.0) | 2 (33.3) | 2 (33.3) | 21 (56.8) |
| Black or African American | 2 (28.6) | 3 (50.0) | 2 (33.3) | 0 (0.0) | 4 (66.7) | 3 (50.0) | 14 (37.8) |
| Asian | 0 (0.0) | 1 (16.7) | 0 (0.0) | 0 (0.0) | 0 (0.0) | 1 (16.7) | 2 (5.4) |
| American Indian or Alaskan Native | 0 (0.0) | 0 (0.0) | 0 (0.0) | 0 (0.0) | 0 (0.0) | 0 (0.0) | 0 (0.0) |
| Native Hawaiian or other Pacific Islander | 0 (0.0) | 0 (0.0) | 0 (0.0) | 0 (0.0) | 0 (0.0) | 0 (0.0) | 0 (0.0) |
| Other | 0 (0.0) | 0 (0.0) | 0 (0.0) | 0 (0.0) | 0 (0.0) | 0 (0.0) | 0 (0.0) |
| Ethnicity, n (%) | | | | | | | |
| Hispanic or Latino | 2 (28.6) | 1 (16.7) | 1 (16.7) | 2 (33.3) | 0 (0.0) | 0 (0.0) | 6 (16.2) |
| Not Hispanic or Latino | 5 (71.4) | 5 (83.3) | 5 (83.3) | 4 (66.7) | 6 (100.0) | 6 (100.0) | 31 (83.8) |
| Height (cm) | | | | | | | |
| n | 7 | 6 | 6 | 6 | 6 | 6 | 37 |
| Mean | 172.06 | 178.18 | 171.38 | 172.25 | 179.23 | 169.33 | 173.69 |
| Standard deviation | 9.816 | 5.347 | 6.502 | 8.454 | 5.395 | 7.686 | 7.841 |
| Weight (kg) | | | | | | | |
| n | 7 | 6 | 6 | 6 | 6 | 6 | 37 |
| Mean | 78.53 | 82.92 | 75.57 | 79.85 | 83.05 | 78.55 | 79.71 |
| Standard deviation | 16.829 | 11.892 | 17.928 | 12.089 | 12.331 | 12.191 | 13.434 |
| BMI (kg/m^2^) | | | | | | | |
| n | 7 | 6 | 6 | 6 | 6 | 6 | 37 |
| Mean | 26.260 | 26.066 | 25.488 | 26.809 | 25.838 | 27.355 | 26.302 |
| Standard deviation | 3.2400 | 3.3675 | 4.4432 | 2.1951 | 3.5873 | 3.2760 | 3.2245 |
| % = 100 🞨 n/N. Baseline was defined as the last measurement prior to the first dose of study drug.  [1]- Percentage was based on the number of female participants. BMI = body mass index. | | | | | | | |

**Table S2.** Dose proportionality analysis results

| **N** | **Dependent variable** | **Estimate**  **(Slope)** | **90% CI** | **Equivalence limits** | **Acceptance Criteria**  **Met** |
| --- | --- | --- | --- | --- | --- |
| **12** | **C_max_** | **1.34** | **1.222 – 1.463** | **0.839 –1.161** | **No** |
| **12** | **AUC_last_** | **1.14** | **1.057 – 1 .233** | **0.839 –1.161** | **No** |
| **12** | **AUC_inf_** | **1.07** | **0.985 – 1.157** | **0.839 –1.161** | **Yes** |

CI: Confidence interval; C_max_: maximum observed drug concentration; AUC_last_: area under the plasma concentration-time curve from time 0 to the last sampling time; AUC_inf_: area under the plasma concentration-time curve from time 0 to infinity

**Table S3.** Results of Two-Way Analysis of Variance (ANOVA) evaluating the effects of dose (50, 100, 200mg), route(IV vs oral), and dose-route interaction on selected pharmacokinetic parameters.

| PK parameter | Dose Effect | | Route Effect | | Dose: Route Interaction | |
| --- | --- | --- | --- | --- | --- | --- |
|  | F value | p-value | F value | p-value | F value | p-value |
| C_max_ | 454.01 | <0.001 | 15.55 | <0.001 | 8.93 | 0.01 |
| AUC_last_ | 543.31 | <0.001 | 0.88 | 0.35 | 0.28 | 0.60 |
| AUC_inf_ | 508.92 | <0.001 | 1.23 | 0.27 | 0.06 | 0.81 |
| Half-Life | 37.64 | <0.001 | 0.32 | 0.58 | 1.18 | 0.28 |
| Vd | 43.18 | <0.001 | 0.70 | 0.41 | 0.01 | 0.93 |
| CL | 2.98 | 0.09 | 0.65 | 0.42 | 0.04 | 0.84 |
| C_max_: maximum observed drug concentration; AUC_last_: area under the plasma concentration-time curve from time 0 to the last sampling time; AUC_inf_: area under the plasma concentration-time curve from time 0 to infinity; CL: clearance; Vd: volume of distribution | | | | | | |

Table S4.	Frequently Reported TEAEs by SOC and PT – Safety Population

| System Organ Class  Preferred Term | 50 mg | | 100 mg | | 200 mg | | Total  (N=37)  n (%) e |
| --- | --- | --- | --- | --- | --- | --- | --- |
|  | Cohort 1  (N=13)  n (%) e | Cohort 2  (N=11)  n (%) e | Cohort 3  (N=12)  n (%) e | Cohort 4  (N=11)  n (%) e | Cohort 5  (N=12)  n (%) e | Cohort 6  (N=12)  n (%) e |  |
| Any frequently reported TEAEs | 1 (7.7) 2 | 1 (9.1) 1 | 6 (50.0) 10 | 4 (36.4) 5 | 7 (58.3) 9 | 6 (50.0) 8 | 17 (45.9) 35 |
| Nervous system disorders | 1 (7.7) 1 | 0 (0.0) 0 | 5 (41.7) 7 | 1 (9.1) 2 | 7 (58.3) 7 | 6 (50.0) 8 | 14 (37.8) 25 |
| Paresthesia | 0 (0.0) 0 | 0 (0.0) 0 | 3 (25.0) 3 | 1 (9.1) 1 | 3 (25.0) 3 | 3 (25.0) 3 | 7 (18.9) 10 |
| Dizziness | 0 (0.0) 0 | 0 (0.0) 0 | 3 (25.0) 3 | 0 (0.0) 0 | 1 (8.3) 1 | 4 (33.3) 4 | 7 (18.9) 8 |
| Headache | 1 (7.7) 1 | 0 (0.0) 0 | 1 (8.3) 1 | 1 (9.1) 1 | 1 (8.3) 1 | 1 (8.3) 1 | 5 (13.5) 5 |
| Somnolence | 0 (0.0) 0 | 0 (0.0) 0 | 0 (0.0) 0 | 0 (0.0) 0 | 2 (16.7) 2 | 0 (0.0) 0 | 2 (5.4) 2 |
| Gastrointestinal disorders | 0 (0.0) 0 | 0 (0.0) 0 | 2 (16.7) 2 | 0 (0.0) 0 | 2 (16.7) 2 | 0 (0.0) 0 | 4 (10.8) 4 |
| Nausea | 0 (0.0) 0 | 0 (0.0) 0 | 1 (8.3) 1 | 0 (0.0) 0 | 1 (8.3) 1 | 0 (0.0) 0 | 2 (5.4) 2 |
| Paresthesia oral | 0 (0.0) 0 | 0 (0.0) 0 | 1 (8.3) 1 | 0 (0.0) 0 | 1 (8.3) 1 | 0 (0.0) 0 | 2 (5.4) 2 |
| General disorders and administration site conditions | 0 (0.0) 0 | 0 (0.0) 0 | 0 (0.0) 0 | 2 (18.2) 2 | 0 (0.0) 0 | 0 (0.0) 0 | 2 (5.4) 2 |
| Vessel puncture site pain | 0 (0.0) 0 | 0 (0.0) 0 | 0 (0.0) 0 | 2 (18.2) 2 | 0 (0.0) 0 | 0 (0.0) 0 | 2 (5.4) 2 |
| Musculoskeletal and connective tissue disorders | 1 (7.7) 1 | 0 (0.0) 0 | 0 (0.0) 0 | 1 (9.1) 1 | 0 (0.0) 0 | 0 (0.0) 0 | 2 (5.4) 2 |
| Back pain | 1 (7.7) 1 | 0 (0.0) 0 | 0 (0.0) 0 | 1 (9.1) 1 | 0 (0.0) 0 | 0 (0.0) 0 | 2 (5.4) 2 |
| Skin and subcutaneous tissue disorders | 0 (0.0) 0 | 1 (9.1) 1 | 1 (8.3) 1 | 0 (0.0) 0 | 0 (0.0) 0 | 0 (0.0) 0 | 2 (5.4) 2 |
| Dermatitis contact | 0 (0.0) 0 | 1 (9.1) 1 | 1 (8.3) 1 | 0 (0.0) 0 | 0 (0.0) 0 | 0 (0.0) 0 | 2 (5.4) 2 |
| A = Single infusion of Topiramate Injection 50 mg; B = Single oral dose of Topamax 50 mg; C = Single infusion of Topiramate Injection 100 mg; D = Single oral dose of Topamax 100 mg; E = Single infusion of Topiramate Injection 200 mg; F = Single oral dose of Topamax 200 mg.  % = 100 🞨n/N. e = number of events.  TEAEs were defined as AEs that started after the first dose of the study drug. AE-reported terms were coded using the MedDRA version 26.1.  AE = adverse event; MedDRA = Medical Dictionary for Regulatory Activities; PT = preferred term; SOC = System Organ Class; TEAE = treatment-emergent adverse event.  Source: Post‑text Table 14.3.1.11 | | | | | | | |

**METHODS**

**Pharmacokinetic analysis**

Plasma samples were collected in K_2_EDTA tubes and centrifuged at approximately 3000 RPM (~ 2000 g) for 10 minutes at 4℃. The plasma samples were then analyzed for topiramate concentrations using a validated liquid chromatography method coupled with tandem mass spectrometry (LC MS/MS) using an AB Sciex API 4000 system. The analytes were separated using a HyperClone BDS C18 column (50 x 2.0 mm, 5µm particle size; Phenomenex) in reverse-phase mode with a 20-90% gradient with a run time of ~4 minutes. The samples were analyzed employing a Multiple Reaction Monitoring mode (MRM), and the transitions monitored were mass to ratio (m/z) 338.1 → 77.9 for topiramate and m/z 350.1 →77.8 for the internal standard (topiramate-d_12_). The calibration curve was linear in the range of 10.0 –10,000 ng/mL. Peak area integrations were performed with Analyst software (version 1.7.1) from Applied Biosystems. Peak area ratios and concentrations were calculated in Watson LIMS software (version 7.6.1). The lower limit of quantification (LLOQ) was 10 ng/mL. The coefficient of variation (%CV) across quality control samples was ≤5.8%, while the relative error (RE) ranged from 2.1–6%. The incurred sample reanalysis (ISR) results showed that 100% of the re-assayed results were within ±20% of the mean results.

The following pharmacokinetic parameters were determined: maximum observed plasma concentration (C_max_), area under the curve from zero to time t (AUC_0-t_), area under the curve from zero to infinity (AUC_0-inf_), half-life (T_1/2_), time to maximum plasma concentration (T_max_), absolute bioavailability (F_abs_), relative bioavailability (F_rel_), clearance (CL), and volume of distribution (Vd). CL was calculated as the administered dose divided by area under the curve from zero to infinity (AUC_0-inf_), with CL reported for IV TPM and apparent clearance (CL/F) reported for oral TPM. Vd was calculated as CL divided by the terminal elimination rate constant (λ_z_), with Vd reported for IV TPM and apparent volume of distribution (Vd/F) reported for oral TPM. The elimination rate constant (λ_z_) was determined from the slope of the terminal log-linear portion of the concentration-time curve. Half-life(T_1/2_) was calculated as In (2)/λ_z_. The absolute bioavailability of the tablet was determined using the following equation:

$$F_{abs}= 100 \times\frac{{AUC}_{oral}\times{Dose}_{iv}}{{AUC}_{iv}\times{Dose}_{oral}}$$

Given that oral topiramate was the reference formulation and IV Topiramate was the investigational formulation, we calculated its relative bioavailability, i.e., a measure of the bioavailability of the IV formulation compared to oral topiramate, using the following equation:

$$F_{rel}= 100 \times\frac{{AUC}_{iv}\times{Dose}_{oral}}{{AUC}_{oral}\times{Dose}_{iv}}$$

Noncompartmental analysis (NCA) was performed using Phoenix WinNonlin version 8.4 (Certara USA Inc., Princeton, NJ, USA)

**Statistical Methods**

Based on data available regarding oral and IV administration of TPM, 36 participants (12 per dose group) were expected to be sufficient to determine the pharmacokinetics, bioavailability, and dose proportionality of Topiramate Injection compared to oral TPM.

The PK Analysis Set was defined as all subjects who provided informed consent, took at least one dose of study drug on Study Day 1, and had at least one post-baseline PK measurement. The safety population was defined as all randomized subjects who received at least one dose of the study drug. Participants who consented to participate and took at least one dose of the study drug were included in the safety analysis.

Categorical data were summarized with counts and percentages of participants. Continuous variables were summarized using descriptive summary statistics, including the number of subjects (n), mean, SD, median, minimum value (min), and maximum value (max) as appropriate. The geometric means, arithmetic means, geometric mean ratios, and 90% confidence intervals (CIs) were calculated for C_max_, AUC_0-inf,_ and AUC_0-t._

To assess dose proportionality (DP), a power model was fitted to log-transformed pharmacokinetic parameters (C_max_, AUC_0-inf,_ and AUC_0-t_) ^20^.

The power model is expressed as:

$PK Parameter= {a \times Dose}^{\beta1}$	Equation 1

$1+ \frac{\log( \theta L)}{log(r)}< \beta1<1+ \frac{log(\theta H)}{log(r)}$	Equation 2

Where PK Parameter refers to the pharmacokinetic parameter of interest, i.e., C_max_, AUC_0-inf,_ or AUC_0-t,_ $a$ represents the baseline constant, β1 represents the slope parameter for dose proportionality, r represents the ratio of the highest (200mg) to lowest (50mg) doses, θ_L_ and θ_H_ represent the lower and higher conventional bioequivalence limits of 0.8 and 1.25 ^21^. The acceptance interval for β1 was calculated to be between 0.839 and 1.161, given the predefined bioequivalence limits (0.8–1.25) and dose ratio (r =4) in Equation 2. DP analyses were done using R (v4.3.1). An exploratory two-way ANOVA was conducted to investigate the effects of dose, route, and dose-route interaction on selected PK parameters.

**Safety Evaluation (Full description)**

Topiramate Injection safety was evaluated by assessing the incidence of treatment-emergent adverse events (TEAEs), serious adverse events, and withdrawals from randomization through the End of Study, as well as changes in clinical laboratory values. The clinical laboratory assessments included hematology, chemistry, urinalysis, and coagulation study values. Further evaluation of electrocardiograms and vital signs was done. Neurologic examination included tests for balance and coordination, vestibular dysfunction, and psychomotor processing speed. Adverse events (AEs) were captured from the date of informed consent through study completion. All AEs were coded to System Organ Class (SOC) and Preferred Term (PT) using MedDRA (version 26.1). For analysis purposes, AEs were classified as pre-treatment AEs or TEAEs, with the former referring to any AE that started before initial dosing of the study drug, and TEAEs were defined as AEs that started after the first dose of the study drug. A Safety Review Committee (SRC) consisting of the CURx Medical Monitor, the Study Principal Investigator, and an external physician with expertise in the use of antiseizure medications reviewed safety and tolerability data for all phases of the study.

**RESULTS**

**Safety**

Topiramate injection was generally well tolerated. As shown in Table 4, TEAE counts (Any TEAEs) were similar between the oral and intravenous dose groups at the 50 mg and 200 mg dose levels, whereas participants experienced more TEAEs following the 100 mg infusion than the 100 mg oral tablet (7 versus 4). Study-related TEAEs were more prevalent in the 200 mg dose. No severe or life-threatening TEAEs were observed for either the oral or intravenous formulations. Four individuals experienced TEAEs with moderate maximum severity: 2 in the 50 mg IV group, 1 in the 100 mg IV group, and 1 in the 200 mg oral group. An overall summary of the frequently reported TEAEs by system organ class is provided in **Table S3**. Nervous system-related TEAEs, which included paresthesia, dizziness, and headache, were the most frequently occurring side effects. Three individuals discontinued the study due to TEAEs. There were two TEAEs in the 50mg IV dose group, i.e., creatinine kinase (CK) levels > 10 times the upper limit of normal, and left lower lobe pneumonia, leading to study discontinuation. These were considered moderate in severity.  The CK elevation was considered possibly related, and the pneumonia was considered unlikely related to the study formulation. The third individual discontinuing the study, who was in the 100 mg IV dose group, had an upper respiratory infection, which was considered mild in severity and not related to the formulation.
